# The Unseen Burden: A Qualitative Investigation of Polish LGBTQ+ Caregivers’ Experiences

**DOI:** 10.3390/jcm14061959

**Published:** 2025-03-14

**Authors:** Magdalena Leszko

**Affiliations:** 1 Department of Psychology, Moravian University, 1200 Main Street, Bethlehem, PA 18018, USA; leszkom@moravian.edu or magdalena.leszko@usz.edu.pl; 2 Department of Psychology, University of Szczecin, Krakowska 69, 71-017 Szczecin, Poland

**Keywords:** Alzheimer’s disease, caregivers, dementia, LGBTQ+

## Abstract

**Background/Objectives**: As the world’s population ages, the growing number of individuals affected by Alzheimer’s disease and related dementias (ADRDs) will undoubtedly continue to impose social and economic challenges. Informal caregivers play a crucial role in providing essential support for individuals with ADRD. However, there is limited research that investigates the psychosocial functioning of caregivers (partners) from minoritized groups. **Methods**: This study aimed to explore the experiences of lesbian, gay, bisexual, transgender and queer (LGBTQ+) caregivers of individuals with ADRDs. Semi-structured in-depth interviews were conducted with seven caregivers of partners diagnosed with ADRDs. Three themes were identified based on reflexive thematic analysis: (i) experiencing familial alienation; (ii) fear about the future; and (iii) finding strength in the face of adversity. **Results**: The research highlighted difficulties reported by LGBTQ+ caregivers, while also showing how such caregivers cope. The findings provide a basis for developing targeted interventions for caregivers from minoritized groups. **Conclusions**: These findings have important implications for policy and intervention development concerning LGBTQ+ caregivers’ mental and physical health outcomes.

## 1. Introduction

Alzheimer’s disease and related dementias (ADRDs) are devastating and progressive neurodegenerative disorders that progressively impair behavioral and cognitive functions, leading to a decline in daily living abilities [1,2]. Age is the most significant risk factor, with the prevalence of ADRDs increasing exponentially after the age of 65 [3]. Globally, 55 million people are currently living with ADRDs, and this number is expected to increase to 155 million in 2050 [4], raising concerns about the economic and societal impact of the disease. Despite extensive research, there is currently no cure for ADRDs. Therefore, the need for informal care is growing.

Although caring for relatives is not a legal obligation in many countries, the majority of individuals depend on close relatives to be able to stay at home [5,6]. Due to rising costs and limited availability of long-term care facilities, informal caregiving remains essential in allowing older adults to age in one place. However, providing care for individuals with ADRDs can be emotionally and physically depleting as the symptoms worsen and impose more responsibilities on the family caregiver over time. A vast amount of research has consistently highlighted the potential negative consequences for caregivers’ physical and mental health, as the demands of caregiving can significantly disrupt various aspects of their lives [7,8,9,10]. Caregivers often experience increased anxiety and depression [11,12], as well as poorer general health and sleep quality [13]. The burden of caregiving can also negatively impact caregivers’ quality of life (QoL) and lead to higher risks of alcohol use, mortality, and morbidity compared to non-caregivers [14,15]. However, it is important to acknowledge that caregivers’ experiences are diverse, and some may derive positive benefits from their caregiving roles and describe their caregiving as rewarding [16].

An increasing number of studies have investigated the physical and emotional demands associated with addressing the needs of an individual with ADRD. However, all data focused mainly on spouses or adult children. To date, few studies have investigated challenges faced by lesbian, gay, bisexual, transgender, and queer (LGBTQ+) individuals who are caregivers of a partner with ADRD. LGBTQ+ individuals are a unique group of caregivers, and it is important to investigate how the caregiving experience affects their well-being because, given the current demographic trends, it is likely that more and more people will assume the caregiver role. Similar to other countries, Poland will also witness an increase in the number of individuals with ADRD. It is estimated that nearly half a million Polish people (7.9% of all individuals aged 65 and older) may currently be affected by dementia. This number is projected to quadruple by 2050 [17,18].

Across the world, older LGBTQ+ adults have experienced systemic discrimination and social stigma throughout their lives, resulting in poorer mental and physical health outcomes compared to their heterosexual counterparts [19,20]. The minority stress theory, which posits that LGBTQ+ individuals experience excess stress due to their stigmatized sexual orientation, has been a key framework for understanding these health disparities [21]. Studies have demonstrated that LGBTQ+ individuals are at an increased risk of poor general health, disability, depression and anxiety, and lower self-esteem and QoL [22,23]. Additionally, they are more likely to engage in adverse health behaviors such as cigarette smoking and excessive drinking compared to heterosexual peers [24].

Research shows that LGBTQ+ individuals attribute their poor mental health to homophobia and prejudice stemming from their different sexual orientations or gender identities [25]. The political climate in Poland has been hostile towards LGBTQ+ individuals for decades. Because of these constraints, many individuals decided to lead double lives and marry other people to avoid rejection and humiliation. One of the examples of how difficult it was for LGBTQ+ people was the so-called “Operation Hyacinth”. It was a secret mass operation in the Polish People’s Republic carried out by the Citizens’ Militia in the years 1985–1987. Its purpose was to create a national database of all Polish homosexuals and people who were in touch with them, and it resulted in the registration of around 11,000 people. The operation led to mass detentions, interrogations, criminal investigations, violence, and incitement to collaborate with the government. Even though the operation took place almost 40 years ago, recent years have witnessed a significant decline in LGBTQ+ rights in Poland (e.g., the creation of public “LGBT-free zones” in approximately 100 cities and towns in Poland). The nationalist politics and rhetoric of the governing Law and Justice Party (PiS) has exerted a negative impact on openness towards the acceptance of LGBTQ+ rights within Polish society [26], with a rise in discriminatory rhetoric and policies targeting the LGBTQ+ community [27]. Given the historical and cultural context of the country, this trend is concerning. A 2021 survey by the European Union Agency for Fundamental Rights (FRA) revealed that approximately 40% of LGBTQ+ individuals in Poland experienced discrimination in the previous 12 months, and 51% avoided seeking help from the authorities due to the fear of discrimination [28].

Taking into account that a significant proportion of individuals within the LGBTQ+ community report poor mental health [21] and are at higher risk of suicide and self-harm compared to heterosexual individuals [29,30], it is important to investigate the experiences of older Polish LGBTQ+ adults who are caregivers and partners of individuals with ADRD. While comparable research exists for developed nations [31,32], Poland lacks studies specifically addressing the challenges of this unique population. To the best of our knowledge, there are no qualitative studies on LGBTQ+ caregivers in Poland. To fill this gap in knowledge, the purpose of this study was to describe and understand the experience of older LGBTQ+ caregivers (partners) of individuals with ADRD in Poland. The adoption of a qualitative methodology was predicated on its ability to yield rich subjective perceptions and personal experiences pertinent to the exploration of this complex phenomenon.

Exploration of the challenges that they face could inform psychological interventions to support caregivers who face discrimination and stigma due to their sexual orientation. Given the heightened prevalence of depression, anxiety, and reduced perceived quality of life among caregivers of individuals with ADRD compared to those caring for individuals with other diseases [33], our study focuses solely on this under-represented population. We also focus only on older caregivers who provide care to their partners because they are more likely to face greater challenges in providing care than adult children and usually reside with the care recipient [34]. Additionally, due to concerns about negative reactions, older adults frequently avoid discussing their sexual needs and experiences, both in general and with healthcare providers [35]. This can result in additional unique challenges that older LGBTQ+ partners of individuals with ADRD face. These findings could have significant implications for policies designed to help caregivers. By understanding how caregivers from minoritized groups cope with caregiving and how these experiences affect their overall well-being, we can develop more effective interventions and resources to ease their burden.

## 2. Method

### 2.1. Participants and Study Procedure

The data for the present study were part of a larger project, running from 2022 to 2024, focused on the experiences, needs, and barriers of Polish individuals caring for their partners diagnosed with ADRD. A qualitative cross-sectional design with semi-structured interviews was used to enable the researchers to gain a deep understanding of the experiences of LGBTQ+ caregivers of partners with ADRD. The interview data were subject to a reflexive thematic analysis to generate themes and interpret the meaning in the data collected. Participation was voluntary, and caregivers were recruited across Poland through advertisements in papers, newsletters, online, social media, and word of mouth. The invitation to participate in the study included a brief overview of the study’s goals, the researcher’s contact information, the risks and benefits of the study, eligibility criteria, time commitment, and a guarantee that their participation would remain confidential. The institutional research ethics board approved the study protocol.

The inclusion criteria for the participation consisted of being the primary caregiver without financial compensation, for at least a year prior to the study, of a partner who had been physician-diagnosed with ADRD, lived in Poland, self-identified as an LBGTQ+ individual, and is 60 years of age or older. All the interviews were conducted online in Polish between December 2023 and August 2024 by the authors and ranged in length from 20 to 80 min. The average length of the interviews was 1 h and 15 min. The interview questions were designed to elicit caregivers’ experiences of their roles and allowed for open-ended discussion about their physical and mental functioning. In order to guarantee anonymity, transcripts were edited to remove the names of participants and other personal information that might identify them. No remuneration was offered for participation in the study.

Each interview was voice-recorded and transcribed. To ensure coding reliability, an iterative, consensus-driven process was employed. Initial independent coding was followed by collaborative discussions to finalize category definitions and mitigate potential bias. Two additional research professionals also reviewed the findings for alternate themes and explanations. The author maintained a reflexive journal throughout data collection and analysis, as qualitative research can be influenced by the researchers’ idiosyncrasies, biases, and experiences. Reflexivity is considered a fundamental methodological practice that allows the re-examination of researchers’ subjectivity and reproduced ideological assumptions throughout the research process [36].

Of the seven LGBTQ+ caregivers who participated in this study, their ages ranged from 60 to 72 years (*M* = 65.6, *SD* = 4.03). Regarding sexual orientation, six participants identified as a gay woman/lesbian, and one participant identified as a gay man. Transgender people were not intentionally excluded; we were unable to recruit these participants for this study. All caregivers identified themselves as unmarried partners of individuals with ADRD (Poland does not legally recognize same-sex unions). The majority of caregivers had been in a relationship with the person living with ADRD for 10 years or more. Four caregivers had master’s degrees, two had bachelor’s degrees, and one caregiver was a high school graduate. In terms of ethnicity, all participants identified as White. All care recipients were diagnosed with dementia due to Alzheimer’s disease.

### 2.2. Data Analysis

A semi-structured interview was developed by the primary researcher. This format allowed the researcher to ask open-ended questions to elicit information about participants’ thoughts, feelings, and challenges in relation to providing care to a partner with ADRD. Interviews were professionally transcribed and deidentified. The interview data were subject to a reflexive thematic analysis to generate themes and interpret meaning in the data collected [37,38]. The process included the researchers familiarizing themselves with the data through reading and re-reading the interviews’ transcripts, coding and categorizing relevant data, identifying potential subthemes and main themes, refining these themes based on the data, and ultimately presenting the final themes with supporting evidence in the report. Potential discrepancies were discussed and resolved through consensus. The quotes from the recordings were translated from Polish into English based on the most recommended methodological approaches for translating in qualitative research [39].

## 3. Results

The three themes focus on a lack of family support, fear about the future, and finding strength in the face of adversity.

### 3.1. Theme 1: Experiencing Familial Alienation

This theme highlights the impact of ADRD on the caregiver’s health and the absence of a familial support system. The disease affected caregivers in several dimensions: physical, mental, social, and economic. While every aspect was mentioned in the interviews, a lack of family support was the most prominent. Symptoms of the disease combined with poor communication with family members confined caregivers to their homes, leading to feelings of isolation.

*Initially, I didn’t mind that my siblings stopped talking to me. It was a relief to finally escape their constant comments. But now… I feel isolated, like a prisoner. I cannot leave her [a partner] home alone. I want to run errands and I would ask my sister for help like other caregivers do, but I can’t. It’s hard.*

Caregivers reported that their contact with family members had been limited ever since the disclosure of their sexual orientation. Some of them never felt accepted by family members but were hoping that it would change with time. Caregivers experienced a sense of sadness when the family knew about their struggles and were still unsupportive

*At first, we were terrified to tell anyone. Then, when we finally did, there was this huge wave of relief that we no longer had to hide. But it’s been hard. My sisters aren’t very good at pretending everything’s okay, and their distance makes me feel like I’ve done something wrong and I’m being punished for it. It’s been devastating. This whole situation has really taken its toll on me.*

### 3.2. Theme 2: Fear About the Future

The majority of caregivers reported high levels of stress and anxiety about the future. The disease presents many challenges to all caregivers, regardless of their sexual orientation. While many caregivers of individuals with ADRD are worried about the progressive nature of the disease and the increasing demands of caregiving [9,20], LGBTQ+ caregivers reported facing fears related not only to their ability to provide adequate care but also to obtain healthcare services. One caregiver, for instance, shared her anxiety about potential discrimination from healthcare providers due to her sexual identity and not being able to obtain medical information.

*Even though we both dreamed of it, marriage was never really possible for us. And now, I worry about the future. What happens if… well, what if something happens to her? It should be me making the medical decisions, not her son who doesn’t even care about her.*

Caregivers were afraid of the unknown and had feelings of uncertainty and insecurity as they were facing an ambiguous future. The demands of caregiving added financial and emotional strain to their worries about their partners’ conditions and their own ability to provide adequate care.

*The nights were the worst. I lie awake crying, feeling completely lost and alone, like something was wrong with me. I keep reading about Alzheimer’s. I’ve already lost a lot of weight—how am I supposed to hold her? What if I get sick? […] Who will stay with her?*

### 3.3. Theme 3: Finding Strength in the Face of Adversity

This theme describes the coping process of LGBTQ+ caregivers of partners with ADRD. To cope with the challenges of the disease and the stigma associated with being a member of the LGBTQ+ community, caregivers developed various coping strategies. Within this theme, it is evident how caregivers, while dealing with the challenges of their role, actively sought ways to alleviate the pressures they faced. They did this mostly by exploring online avenues for support or relying on friends.

*I once heard that friends are God’s way of making up for our families. And we’re truly lucky because we have the most amazing friends. They’ve become my family, really. With them, I don’t feel judged.[…] It gives me so much hope to see more and more people openly talking about their sexual orientation these days. It makes me feel like society is finally moving towards a place where I can truly be myself.*

Another caregiver highlighted the value of online support groups, especially in overcoming the social isolation and time constraints that often accompany caregiving.

*We’re like a little team. Thanks to the support group, I’ve formed friendships that have been really helpful. Only another caregiver can understand the day-to-day struggles and the frustrations.*

## 4. Discussion

As the world’s population ages, the demand for long-term care services is increasing. Traditionally, families, especially spouses or adult children, have become primary caregivers for their disease-affected relatives. The literature indicates a connection between providing care for a person with Alzheimer’s disease or related dementia and one’s well-being, suggesting that there are positive and negative consequences of providing care that influence caregivers’ mental and physical health. However, significant gaps remain in our understanding of how being a member of a minoritized group affects caregivers’ experience. Therefore, the objective of this study was to examine common challenges encountered by LGBTQ+ caregivers and to investigate the methods they employ to address these challenges.

Seven semi-structured interviews with LGBTQ+ caregivers who provide care to a partner diagnosed with ADRD were analyzed. Three dominant themes emerged in this study: experiencing familial alienation, fear about the future, and finding strength in the face of adversity. Some caregivers have faced criticism from family members ever since they disclosed their sexual orientation. This led to strained relationships and reduced contact. A lack of support combined with the ongoing stress of providing care can diminish the quality of care they provide and heighten their risk of burnout. This theme is of great importance because research shows that strong family ties are essential for well-being, especially later in life when people may face increased isolation and require more support [40,41]. The findings emphasize the need to understand the loneliness experienced by caregivers, especially those from minoritized groups, because they are at higher risk of having reduced contact with their family members. This can significantly impact their mental and physical health as loneliness has been linked to severe health issues like depression, heart disease, reduced quality of life, and suicidal ideation [42,43]. This study found that caregivers experienced significant stress related to various aspects of their loved one’s illness. This stress was compounded for LGBTQ+ caregivers in Poland due to the lack of legal recognition for same-sex couples. Without legal protections, these caregivers may be denied access to medical information and decision-making regarding their partners, creating additional anxiety and challenges. Importantly, our study also highlighted the vital role of online support groups in providing information and emotional support to LGBTQ+ caregivers. Furthermore, caregivers emphasized the invaluable support of friends in helping them cope with stress and feelings of exclusion.

Taken together, this study contributes to the literature on both minoritized groups and caregiving. LBGTQ+ caregivers face unique challenges beyond those experienced by other caregivers. Being a member of a minoritized group while caring for a partner can lead to feelings of isolation and judgment. In Poland, a high percentage of individuals with ADRD receive care at home from family members, reflecting a strong cultural tradition of family caregiving. However, the demands of caregiving combined with stigmatization and discrimination can lead to significant caregiver burden, physical and mental exhaustion, and feelings of isolation. It is important to note that the constant demands of caregiving can quickly lead to caregiver burden and negatively impact the quality of care provided [44].

By conducting interviews, the study provides important new information about caregiving that could not be ascertained from studies employing caregivers from non-minoritized groups. However, there are several limitations to this study that should be addressed in future research. First, participants in this sample consisted of a homogenous group (well-educated White caregivers) and were mostly women. This is consistent with previous research, which has shown that the role of the informal caregiver is disproportionately occupied by women [45]. Additionally, women seem to be more likely than men to participate in studies [46]. The study’s results represent the views of older LGBTQ+ caregivers who opted to participate, thereby introducing a selection bias that may limit the generalizability of the findings. Future research should include more non-binary participants to better understand how LGBTQ+ individuals experience caregiving differently. The semi-structured interview format, while helpful, may have limited the exploration of certain caregiving experiences. Additionally, the sample size was small, and all participants were Polish. Consequently, the findings may not be transferable to LGBTQ+ caregivers from other countries. Future research needs to recruit a larger and more diverse population of LGBTQ+ caregivers of partners with ADRD to gain a deeper understanding of the challenges faced by caregivers.

In conclusion, LGBTQ+ Polish caregivers of individuals with ADRD face a unique set of challenges, including potential conflicts with family and friends who may not accept their sexual orientation. However, they also find strength and support within their communities. This paper sheds light on the difficulties LBGTQ+ caregivers encounter but also demonstrates how they cope with those challenges. The results also emphasize the need to develop care interventions to better support caregivers that would reduce stigma and the symptoms of caregiver burden. Interventions are essential for this group, as caregivers, apart from caregiver burden, often face additional burdens due to discrimination. Ultimately, government support, increased awareness, and social campaigns are crucial for ensuring the well-being of LGBTQ+ caregivers.

## Data Availability

The dataset used and analyzed during the current study is available from the corresponding author upon request.
